# A Multidisciplinary Approach to Anesthetic Management of a Parturient with Severe Aortic Stenosis

**DOI:** 10.1155/2015/489157

**Published:** 2015-05-21

**Authors:** Kalpana Tyagaraj, David A. Gutman, Lynn Belliveau, Adnan Sadiq, Alok Bhutada, Dennis E. Feierman

**Affiliations:** ^1^Department of Anesthesiology, Maimonides Medical Center, 4802 10th Avenue, Brooklyn, NY 11219, USA; ^2^Department of Cardiology, Maimonides Medical Center, 4802 10th Avenue, Brooklyn, NY 11219, USA; ^3^Department of Neonatology, Maimonides Medical Center, 4802 10th Avenue, Brooklyn, NY 11219, USA

## Abstract

In order to optimize anesthetic management and avoid adverse maternal and fetal outcomes, a clear understanding of the changes in cardiovascular physiology that occur during pregnancy is paramount. The effects of normal gestation on the cardiovascular system are particularly significant in a parturient with cardiac valvular pathology. We present a case of a 27-year-old G2P0 at 37 weeks with a past medical history of diabetes, macrosomia, congenital bicuspid aortic valve with severe stenosis (valve area 0.7 cm^2^) who was scheduled for elective C-section. A multidisciplinary discussion involving cardiologists, cardiac surgeons, obstetric surgeons, neonatal intensivists, perfusion staff, anesthesiologists, and nursing staff was held to formulate a plan for the perioperative management of this parturient. Also, contingency plans were formulated and discussed with the care providers, in the event of acute decompensation of the mother and baby and possible need for emergency aortic valvuloplasty and/or aortic valve replacement.

## 1. Introduction

Anesthetic management of parturients with significant cardiac valvular pathology can be challenging. Marked hemodynamic changes during normal pregnancy account for signs and symptoms that can mimic those of heart disease (Table 2). The cardiovascular changes of normal pregnancy, including increased hear rate, stroke volume, cardiac output, increased circulating blood volume, and decreased systemic vascular resistance, place the myocardium under stress. This is well tolerated in a healthy young parturient but often compromised myocardium can fail, placing the patient and her fetus at risk. The myocardium can fail any time during the antepartum, intrapartum, and postpartum periods. Parturients with cardiac valvular disease have greater mortality and morbidity compared to their healthy counterparts without cardiac disease. The incidence of cardiac disease in pregnancy has remained stable (0.1–4%) [1]. Though rare, nonobstetrical maternal death as a result of cardiac disease accounts for approximately 15% of all maternal obstetrical mortality. Peripartum maternal morbidity for parturients with aortic stenosis and New York Heart Association (NYHA) functional classes III and IV symptoms is 10% [2] as compared to 0.4% for those in NYHA functional classes I and II.

## 2. Case Presentation

A 27-year-old G2P0 with a past medical history of insulin dependent diabetes (since the age of 9) and congenital bicuspid aortic valve presents at 37 weeks' gestation to the Labor and Delivery Suite. On physical exam it was noted that she had a grade 4 mummer at the right sternal boarder and +2 bilateral edema. She denied shortness of breath, dyspnea on exertion, or chest pain. Fourteen months before presentation, her valve area was 0.9 cm^2^. She presented at this time for poorly controlled diabetes and fetal macrosomia for induction of labor. Secondary to her history of bicuspid aortic valve with an area that was 0.9 cm^2^, she was sent to the cardiothoracic intense care unit for monitoring. A transthoracic echo revealed an even more stenotic valve with an area of 0.7 cm^2^, with an estimated peak gradient of 64 and mean gradient of 38. A multidisciplinary discussion was held with interventional cardiologists, cardiac surgeons, obstetric surgeons, neonatal intensivists, perfusion staff, anesthesiologists, and nursing staff. A C-section was planned.

**Figure 1:**
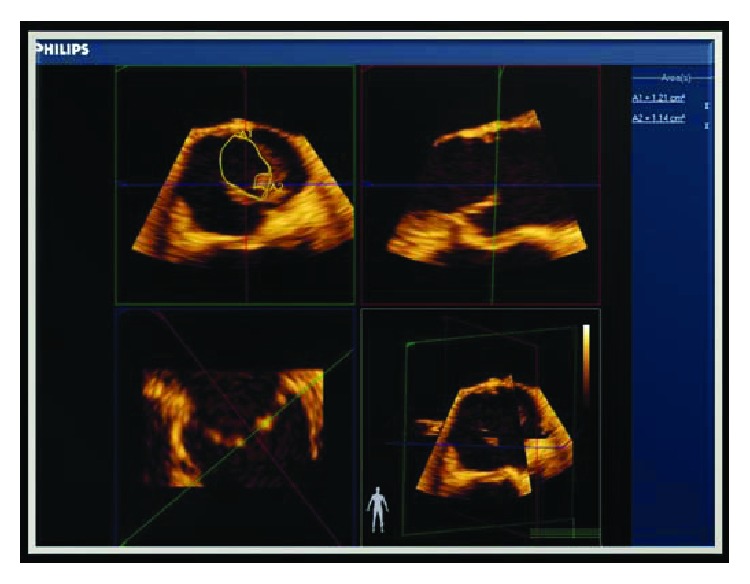
TEE 3D image (Philips Qlab) of the aortic valve using the planimetry method to measure the aortic valve area. TEE = Transesophageal echocardiography.

**Figure 2:**
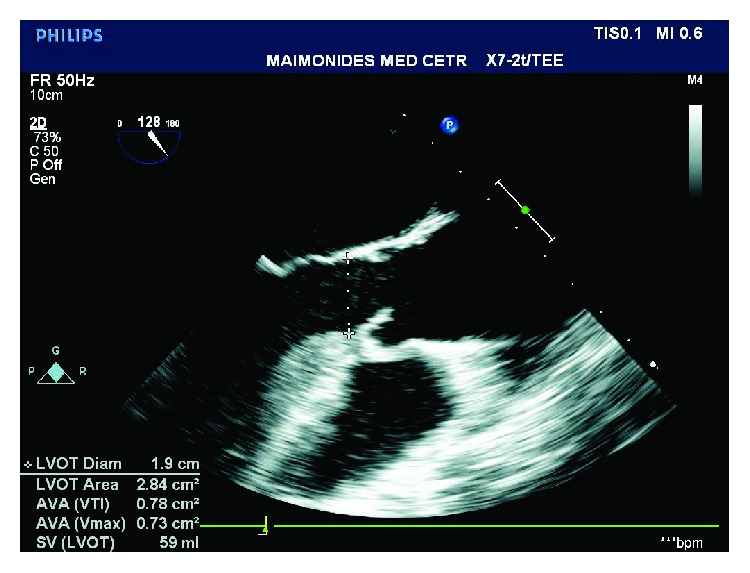
TEE image of the midesophageal aortic valve long axis (ME AV LAX) view measuring the LVOT diameter (one of three measurements used to calculate the aortic valve area using the continuity equation). LVOT = left ventricular outflow tract.

**Figure 3:**
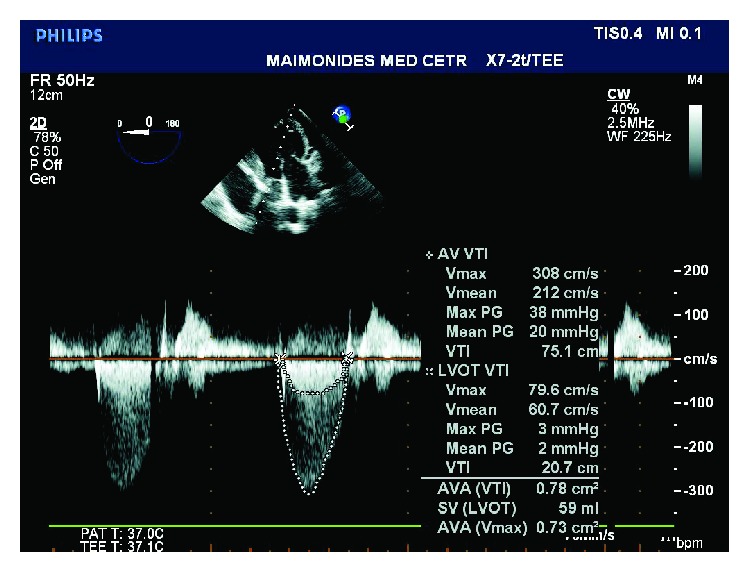
TEE image of the deep transgastric long axis (Deep TG LAX) view measuring the gradient through the aortic valve and LVOT (two of three measurements used to calculate the aortic valve area using the continuity equation).

The patient received 30 mL of nonparticulate antacid orally and metoclopramide 10 mg intravenously 30 minutes prior to the planned procedure. The patient was brought to the hybrid operating room. Standard ASA monitors were applied. Two large bore antecubital IVs (18 g in the right upper extremity and 16 in the left upper extremity) and left radial arterial line were placed. Induction was initiated with two 1 mg boluses of Midazolam, followed by a remifentanil infusion started at 0.3 mcg/kg/min. Esmolol 20 mg boluses were used to titrate the heart rate to a target heart rate of 70–80. A rapid sequence induction was performed with a combination of 14 mg of etomidate, 80 mg of propofol, and 120 mg succinylcholine and the airway was secured. Maintenance of anesthesia consisted of remifentanil infusion and sevoflurane 2%. Midazolam 2 mgs and fentanyl 100 mcgs were administered after the delivery of the baby. The baby was delivered within 3 minutes of intubation. A transesophageal echo probe was inserted. Intraoperative TEE findings were aortic stenosis with 0.8 cm^2^ valve area (Figures 1 and 2), mild AI, mild MR, peak transaortic valvular gradient of 38 and mean transaortic valvular gradient of 20 (Figure 3), and mild pulmonary valve regurgitation. The rest of the intraoperative course was unremarkable. The mother was successfully extubated at end of the procedure with careful titration of a beta-blocker for heart rate and blood pressure control (Table 2). The patient was transferred to cardiothoracic intensive care unit (CTICU) for close monitoring. Postoperative pain management consisted of IV PCA hydromorphone, intravenous ketorolac, and intravenous acetaminophen. The postoperative course was uneventful and the patient was discharged on post-op day 3. The patient returned 11 months later and underwent successful minimally invasive aortic valve replacement.

The NICU staff was present for the delivery and the baby was taken to the NICU for close monitoring. The initial APGAR score was 2 and the baby was floppy and apneic. After initial positive pressure ventilation, the baby was intubated. APGAR scores quickly improved to 5 and then 8 at which point the baby was extubated. The baby was observed in the NICU for a few days with an initial diagnosis of respiratory distress. The baby was treated with C-PAP for one day and the diagnosis of apnea and transient tachypnea of the newborn baby of a diabetic mother was made. Chest X-ray was unremarkable and, after one day on IV glucose solutions, the baby was able to maintain normal glucose levels. The apnea improved and the baby was discharged to the nursery on day 4.

## 3. Discussion

Aortic stenosis is typically seen in the elderly population; when encountered in the younger population, it is usually attributed to congenital bicuspid aortic valve. Maternal and perinatal mortality of 17% and 32% respectively have been reported in parturients with severe aortic stenosis. During pregnancy, a parturient undergoes many physiologic changes to best prepare for the needs of the growing fetus and the placenta. One of the biggest changes during pregnancy is the increase in cardiac output secondary to an increase in heart rate and stroke volume, up to a 75% increase above prelabor values (Table 1). These normal physiological changes of pregnancy can precipitate heart failure in patient with severe AS. The increase in cardiac output and blood volume attributable to uterine contractions during labor, in face of the fixed stroke volume of aortic stenosis, may precipitate tachycardia. Tachycardia is worsened further by the pain-induced sympathetic stimulation and acute decompensation can set in.

## 4. Obstetric Management of a Parturient with Aortic Stenosis

Patients who are symptomatic or who have a peak outflow gradient of more than 50 mm Hg are advised to delay conception until after surgical correction. Termination of pregnancy should be strongly considered if the patient is symptomatic before the end of the 1st trimester. Aortic valve replacement and palliative aortic balloon valvuloplasty have been performed during pregnancy with associated maternal and fetal risk. Vaginal delivery is preferred, facilitated by instrumental delivery to avoid hemodynamic changes associated with the Valsalva maneuver. The option of Cesarean Section is reserved for maternal and fetal indications.

## 5. Anesthetic Management of a Parturient with Aortic Stenosis

It is controversial whether general or neuraxial anesthesia is more appropriate for parturients with aortic stenosis. The choice should not be made depending on the severity of stenosis or the transvalvular gradient, rather the preanesthetic assessment of the symptoms and signs, right and left heart function, function of the other cardiac valves, and presence or absence of pulmonary hypertension. Hemodynamic monitoring with A-line and CVP/PAC may be indicated.

Epidural analgesia is ideal for labor pain management since it provides adequate pain control and also minimizes blood volume and cardiac output increases after delivery. It is prudent to avoid epinephrine “test dose” and careful titration of dosing the epidural catheter to avoid sudden decreases in SVR is essential. Dilute local anesthetics with opioids are preferred to minimize sympathectomy and the decrease in SVR.

If Cesarean Section is indicated in a patient with severe aortic stenosis, general anesthesia has traditionally been advocated and remains the gold standard. There are published case reports which have described administration of neuraxial techniques successfully; publication bias cannot be ruled out. Goals of anesthetic management are as follows:

1. Maintenance of a normal heart rate and sinus rhythm: since patients with severe AS do not tolerate bradycardia and can decompensate with tachycardia, the overall anesthetic consideration with respect to heart rate is to maintain a normal to slightly elevated heart rate. Patients with severe AS can decompensate with tachycardia, since the noncompliant, hypertrophic ventricle needs time to fill and the increased heart's demand for oxygen associated with tachycardia in the face of limited supply secondary to decreased diastolic time (and coronary perfusion time) can result in myocardial ischemia which can increase LVEDP and even further decrease myocardial perfusion. Development of atrial fibrillation with rapid ventricular response decreases the diastolic filling time and eliminates the atrial component of left ventricular filling. Prompt restoration of sinus rhythm is vital to avoid hypotension or ventricular failure.
2. Maintenance of intravascular volume and preload: the stroke volume, hence cardiac output, is maintained if the end diastolic volume is maintained via adequate venous return.
3. Maintenance of systemic vascular resistance: decreases in systemic vascular resistance, typically found in pregnancy, can lead to decreased myocardial perfusion that, coupled with ventricular hypertrophy, can result in myocardial ischemia leading to left ventricular failure. Careful, close monitoring of blood pressure with an arterial line and heart rate is paramount.
4. Avoidance of myocardial depression: agents like propofol and thiopental are to be avoided to minimize myocardial depression.
5. Avoid aortocaval compression.

Opioid based induction and maintenance are preferred to achieve and maintain the hemodynamic goals. Potential fetal depression warrants the immediate presence of neonatal resuscitation team.

Careful planning using a multidisciplinary approach is essential for optimal maternal and neonatal outcomes. The key to manage this patient was to control the heart rate, thus decreasing the heart's demand for oxygen in the face of limited supply secondary to the ramifications to the aortic valve stenotic pathology.

## Figures and Tables

**Table 1:** Hemodynamic changes associated with pregnancy.

Cardiovascular changes	% change	Implication for severe AS
Blood volume	↑30–40%	Potential volume overload
Plasma volume	↑40%	
Red cell mass	↑30%	
Hematocrit	↓29–34%	↓ oxygen delivery
Blood pressure	↓20%	↓ coronary perfusion
Systolic	↓5%	
Diastolic	↓15%	
SVR	↓15%	↓ coronary perfusion
CVP pressures	↓30%	
PA pressures	↓30%	
Heart rate	↑15–20%	↓ coronary perfusion time
Stroke volume	↑15–20%	Fixed in AS→↓↓ in BP

EKG: electrocardiogram; SR: sinus rhythm; FHR: fetal heart rate.

**Table 2:** Intrapartum and postpartum hemodynamic measurements.

	Before induction 8:45	Induction 9:40	Intubation 9:44	Delivery 9:46	Postpartum		
					After 15 min 9:51	Extubation 11:06	CTICU 11:38
Blood pressure (mmHg)	149/90	136/92	103/75	120/79	111/74	148/104	153/87
Heart rate (beats/min)	94	83	80	75	69	93	84
SpO_2_ (%)	100	100	100	100	100	100	100
EKG	SR	SR	SR	SR	SR	SR	SR
FHR (beats/min)	130	130					
FHR tracing category	I	I					

## References

[1] van Mook W. N., Peeters L. (2005). Severe cardiac disease in pregnancy, part I: hemodynamic changes and complaints during pregnancy, and general management of cardiac disease in pregnancy. *Current Opinion in Critical Care*.

[2] http://www.clevelandclinicmeded.com/medicalpubs/diseasemanagement/cardiology/pregnancy-and-heart-disease/#assessment.

